# Horizontal Synchronization of Neuronal Activity in the Barrel Cortex of the Neonatal Rat by Spindle-Burst Oscillations

**DOI:** 10.3389/fncel.2018.00005

**Published:** 2018-01-19

**Authors:** Dmitrii Suchkov, Lyaila Sharipzyanova, Marat Minlebaev

**Affiliations:** ^1^Laboratory of Neurobiology, Kazan Federal University, Kazan, Russia; ^2^INMED-INSERM U901, Aix-Marseille Université, Marseille, France

**Keywords:** early gamma oscillation, spindle-burst, immature cortical activity, development, neonatal rat, barrel cortex

## Abstract

During development, activity in the somatosensory cortex is characterized by intermittent oscillatory bursts at gamma (early gamma-oscillations, EGOs) and alpha–beta (spindle-bursts, SBs) frequencies. Here, we explored the topography of EGOs and SBs in the neighbor barrels of the whisker-related barrel cortex of neonatal rats (P4-7) during responses evoked by simultaneous activation of multiple whiskers as it occurs during natural conditions. We found that brief simultaneous deflection of all whiskers evoked complex neuronal responses comprised of EGOs and SBs. In contrast to EGOs, that specifically synchronized neuronal activity in each individual barrel, SBs efficiently synchronized activity between neighboring barrels. After plucking a single whisker, synchronous stimulation of spared whiskers evoked EGO-lacking responses in the whisker-deprived barrel, even though the remaining neuronal activity was synchronized by SBs in neighboring barrels. Thus, EGOs specifically support topographic synchronization of neuronal activity within barrels, whereas SBs support horizontal synchronization between neighboring barrels during stimulation of multiple whiskers. We suggest that these two co-existing activity patterns coordinate activity-dependent formation of topographic maps and support the emergence of integrative functions in the primary somatosensory cortex during the critical period of somatosensory maps development.

## Introduction

Spontaneous and sensory-evoked neuronal activity is critical for the development of the cortical maps in the somatosensory system (Van der Loos and Woolsey, 1973; Sieben et al., 2013); for review see (Fox, 1995; Hanganu-Opatz, 2010; Kilb et al., 2011; Luhmann and Khazipov, 2017). Immature cortical network activity is organized by oscillatory bursting activity patterns, expressed specifically during the critical period for somatotopic maps formation that spans the first postnatal week in rodents. Based on the dominant oscillatory frequency, this oscillatory activity is divided in two categories: early gamma oscillations (EGOs) with predominant activity in the gamma frequency band (30–60 Hz) and spindle-bursts (SBs) with predominant frequencies at 5–25 Hz (Khazipov et al., 2004; Hanganu-Opatz et al., 2006; Minlebaev et al., 2007; Yang et al., 2009; Tiriac et al., 2012; Gerasimova et al., 2014). Both EGOs and SBs are internally generated activity patterns that persist after sensory deafferentation and are reliably evoked by passive sensory stimulation and sensory feedback from movement (Khazipov et al., 2004; Akhmetshina et al., 2016). EGOs and SBs are not mutually exclusive patterns and may coexist during spontaneous activity and sensory-evoked responses. Despite the numerous similarities between EGOs and SBs (see for review Luhmann and Khazipov, 2017), there are some differences between these two activity patterns. First, EGOs are more prominent during evoked responses by strictly topographic sensory input, such single principal whisker (PW) stimulation in the corresponding barrel of whisker-related S1 barrel cortex (Minlebaev et al., 2011). In contrast, SBs predominate during evoked responses from multiple inputs, such as electrical stimulation of the entire whisker pad or mechanical stimulation of the hindlimb and forelimb (Khazipov et al., 2004; Minlebaev et al., 2009). Second, SBs occupy larger cortical territories (Khazipov et al., 2004, see Supplementary Figures S3, S4; Minlebaev et al., 2007), while EGOs are mainly restricted to one barrel (200–300 microns in diameter) (Yang et al., 2009; Mitrukhina et al., 2015). Finally, mimicking EGOs and SBs in neonatal thalamocortical slices induces opposing plasticity mechanisms (LTP or LTD) at thalamocortical synapses on L4 neurons (Minlebaev et al., 2011). These observations indicate that these two activity patterns may play different roles in orchestrating cortical activity in the neonatal cortex during the critical period.

Here, we examined the roles of EGOs and SBs in synchronization of neuronal activity within and between neighboring barrels of neonatal rat barrel cortex during evoked responses by synchronous stimulation of all whiskers as it occurs under natural conditions during spontaneous whisker movements and passive whisker stimulation (Akhmetshina et al., 2016). We observed that simultaneous whisker stimulation evoked complex responses containing both EGOs and SBs. We found that EGOs specifically synchronized neuronal activity within each individual barrel but not between barrels and that SBs supported horizontal synchronization between neighboring barrels. Horizontal interbarrel SB synchronization also persisted in the acute sensory deprived barrel. Thus, gamma synchronization of neuronal activity within barrels, which has been previously shown during evoked responses by single whisker stimulation, is also present during the responses evoked by stimulation of multiple whiskers, and horizontal synchronization between barrels is supported by SBs likely reflecting an emergence of integrative functions in the barrel cortex.

## Materials and Methods

### Ethics Statement

All animal-use protocols followed the guidelines of the French National Institute of Health and Medical Research (INSERM, provisional approval N007.08.01) and the Kazan Federal University on the use of laboratory animals (ethical approval by the Institutional Animal Care and Use Committee of Kazan State Medical University N9-2013).

### Surgery

Male and female Wistar rats from postnatal day [P] 4–7 (15 rat pups) were used. The surgery was performed under isoflurane anesthesia (5% for the induction and 1.5% during the surgery). After an incision to remove the scalp, the skull was cleaned and covered with dental cement (grip cement) except for a 4–9 mm^2^ window above the barrel cortex for electrode placement. Animals were warmed and left to recover from anesthesia. During recordings, the head was fixed to the frame of the stereotaxic apparatus by ball-joint bars. Animals were surrounded by a cotton nest and heated via a thermal pad (35–37°C). A chloride-coated silver wire was placed in the cerebellum or visual cortex and served as the ground electrode. All recordings were made from rat pups anesthetized by i.p. injection of urethane (1 g/kg).

### Stimulation

Before the experiment, whiskers were trimmed to a length of a 3 mm. Whiskers were stimulated by piezo deflection or air-puff. For piezo whisker stimulation, the needle (30G) was glued to the end of piezo benders (Noliac). The tip of the whisker was inserted 2 mm into the blunt tip of the needle so that the whisker rested snugly inside. To induce deflection of the piezo benders, square 80–90 V pulses of 10 ms duration were applied. For air-puff, a short puff of air (10 ms) with 20 psi pressure was applied to the whisker/-s through the plastic tube (1 mm diameter). To avoid depression of the evoked response, whiskers were stimulated every 30 s. The cortical response was evoked using three stimulation protocols. (1) “Principal whisker” (PW) was the stimulation protocol with piezo- or air-puff deflection of single PW that projected to the recorded barrel. (2) In the “all whiskers” stimulation protocol (PW+AWs), the air-puff was applied to a group of whiskers, including those that projected to and around the recorded barrels. (3) “Adjacent whiskers” (AWs) stimulation protocol involved air-puff stimulation of all whiskers, except the PW, that had been carefully pulled out (*n* = 8, P4-7) or covered by the plastic shield (*n* = 7, P4-7) prior to stimulation. We have compared the MUA in the sensory deprived barrel in the experiments with pulled out or covered whisker. Results showed no difference in the latency (46 ± 11 and 46 ± 9 ms, correspondingly) or rate of the MUA (15 ± 12 and 15 ± 8%, respectively) following the ‘AWs’ stimulation (**Supplementary Figures S1B,C**), thus the results of two sensory deprivation protocols were pooled together.

### Intrinsic Signal Optical Imaging

Optical imaging of Intrinsic Signal (OIS) was recorded using a video acquisition system. The camera was positioned orthogonally to the exposed skull above the estimated location of the somatosensory cortex. To detect the OIS, the camera was focused at 400–1200 μm (depending on animal age) below the skull to the expected depth of the thalamorecipient layer of the barrel cortex. To achieve equal illumination, three red (Arlight, 610 nm, 3 W, China) highlighting diodes were placed around the animal’s head. The reflected light was collected by the CCD camera (QICAM Fast 1394, 130 × 174 resolution, 1 pixel = 35 μm). For detection of the cortical positions of two barrels used for the characterization of the evoked electrical neuronal activity by different stimulation types, two longest whiskers from the back of the snout (from B–E rows) were simultaneously stimulated. Because of the low amplitude, the OIS was calculated based on the average of 20 repeated video acquisitions. Each repetition had a duration of 60 s with 5 s of a pre-stim period followed by the 2 Hz train of whisker(s) deflections (10 ms duration each) during 10 and 45 s of recovery. During preprocessing, the recorded video was downsampled to 10 Hz, followed by OIS detection when an averaged intra-stimulus video frame was compared to an averaged pre-stimulus video frame. OIS detection was performed manually by the operator by masking off the area with decreased light intensity, followed by automated estimation of OIS size by calculating the angular averaged intensity around the center of the user-defined OIS region. All values from the reference region that exceeded the significance level of 2.5 Jackknife standard deviation (SD) of noise were considered as the OIS [for details of the OIS denoising and detection (see Sintsov et al., 2016)]. For better visualization, all OISs were inverted, which indicated that the decrease in reflected light intensity had positive changes throughout the manuscript.

### Electrophysiological Recordings

Extracellular recordings of evoked cortical activity in the barrel cortex were performed using two types of multisite silicon probes (Neuronexus Technologies, United States): (1) 4 × 4 16-channel probes with a separation distance of 200 μm between shanks and 200 μm between electrodes in each shank and (2) 4 × 8 32-channel probes with a 200-μm between shanks and a 50–200 μm distance between electrodes in each shank. The electrodes were placed into the barrels localized using OIS imaging as described above. The recording electrode was aligned perpendicular to the skull surface to reach the barrels of interest. The electrode was placed at the depth of the granular layer of the sensory-recipient barrel that was confirmed by the combination of followed factors: (i) presence of a short latency evoked local field potential (LFP) deflection and multiple unit activity (MUA) following the stimulation of the corresponding whisker (Minlebaev et al., 2009; Yang et al., 2013; Mitrukhina et al., 2015) and (ii) predominance of gamma frequency in the evoked LFP and MUA (Minlebaev et al., 2011). The analysis was done based on recordings from 20 to 100 cortical responses. The signals were amplified (× 10,000), filtered (0.1 Hz–10 kHz) using a 128-channel amplifier (Neuralynx, United States) or 64-channel amplifier (DIPSI, France) and analyzed *post hoc*. Because of the different whisker lengths and corresponding barrels size in the barrel cortex, we preferentially recorded evoked activity in the first barrels (1–3) of the rows (B–E). Barrels from different arcs and rows were preferentially recorded. For definition between the recorded barrels, we called the barrel, receiving the topographical sensory input during the stimulation, as ‘principal barrel’ (PB) while its neighbors were named as ‘adjacent barrels’ (AB). However, it is important to note, that during ‘PW+AWs’ and ‘AWs’ types of stimulation, all stimulated barrels received the topographical sensory input from the corresponding whiskers.

### Data Analysis

Raw data were preprocessed using custom-written functions in MATLAB (MathWorks). Briefly, raw data were explored to detect MUA, followed by raw data downsampling to 1000 Hz for further analysis of LFP. The analysis was conducted in 500 ms windows: (1) after the stimulus to define evoked activity and (2) before the stimulus to characterize spontaneous activity to estimate the baseline. MUA was detected at a band-passed signal (>200 and <4000 Hz) when all negative events exceeding 3.5 SD were considered as spikes (>99.9% confidence) (Mitrukhina et al., 2015). Because of a high level of MUA variation between the animals, the power of the MUA in different stimulation conditions was normalized to MUA response to PW stimulation in the animal.

Spectral features of MUA and its coherency to LFP were calculated using the Chronux toolbox functions (Bokil et al., 2010). This toolbox was used because of overcame some limitations of conventional Fourier analysis by the Slepian function use that resulted in a better concentration of the frequency peak energy. However, in contrast to PW and PW+AWs stimulation types, where used MUA and LFP were from the same barrel, coherence during AWs stimulation was calculated using MUA from the sensory deprived barrel and the LFP from the recorded adjacent barrel. To exclude the coincidence of coherence coefficients, the random distribution was established using multiple shuffling of MUA timestamps, followed by recalculation of coherence coefficients. Shuffle-based coherence coefficients were considered as a reference and used to calculate the significance threshold. We also analyzed MUA phase locking to the LFP from the same and AB at different frequencies. For that, the LFP from the barrel of interest was first filtered using continuous time-frequency analysis. Morlet mother wavelet was applied to LFP consistently with the frequency increment of 1 Hz starting from 5 to 100 Hz. Secondly, Hilbert transform of filtered data was used to extract instantaneous phases of the LFP. Each spike phase was defined from filtered LFP according to its timestamp. The significance of the MUA phase lock was calculated using estimated angles of randomly distributed MUA versus its LFP. The threshold of significance was set at 2.5 Jackknife standard deviation + mean value of the results calculated on shuffled data (100 iterations). Jackknife standard deviation was calculated using the Jackknife method provided in the Chronux toolbox. The reliable peaks (for coherence and phase lock analysis) were defined as peaks exceeding the threshold and consisted of the more than two interlinked points. Finally, the event synchronization algorithm (Quian Quiroga et al., 2002) was used to calculate synchronicity of oscillatory responses between cortical columns. Briefly, the periods of synchronicity were defined as the quasi-simultaneous appearances of the AB MUA in the 5 ms window centered by the PB spikes. The significance threshold was set at 2.5 SD + mean value of the synchronicity calculated using multiple shuffling (100 iterations). Only events exceeded the threshold were taken into the account and used for further analysis. The confidence interval (CI) was calculated by using 2.5 Jackknife standard deviation that corresponds to the significance threshold of 0.05.

## Results

### Barrel Responses Evoked by Multiple Whisker Stimulation

**FIGURE 1:**
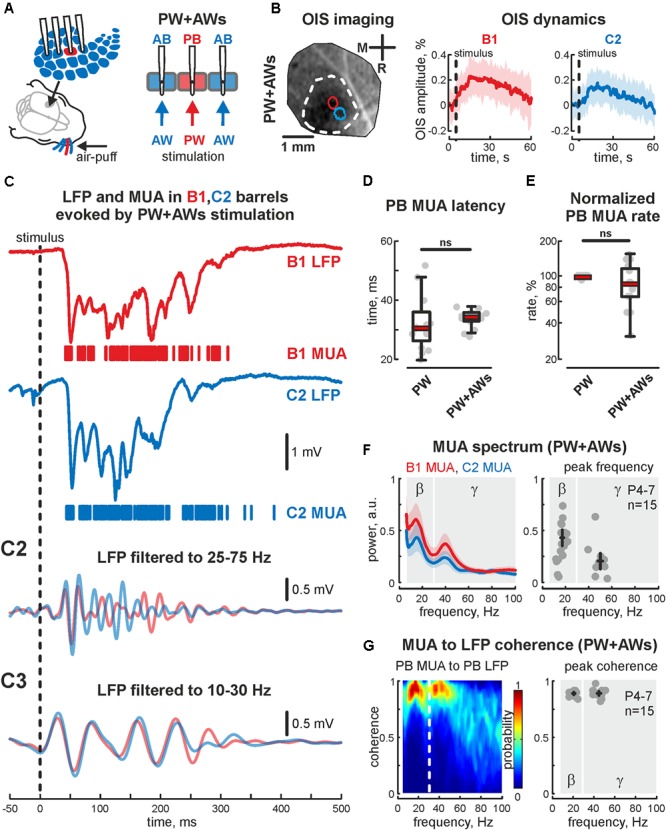
Cortical responses from the neonatal rat barrel system evoked by PW+AWs stimulation. **(A)** Scheme of the experimental setup with multishank recordings of the evoked activity from barrels receiving sensory input during all whiskers stimulation (PW+AWs), the PB with corresponding PW are shown by red, the neighboring ABs are blue. Note, that during ‘PW+AWs’ stimulation all barrels received the sensory input from their whiskers. **(B)** OISs evoked in barrel cortex (left) and OIS dynamics in B1 (red) and C2 (blue) barrels (right) are shown. Confidence interval (CI) is shown by the shaded area. **(C)** Evoked LFP (trace) and MUA (bars) recorded in B1 (red) and C2 (blue) barrels. Bandpass filtered B1 and C2 LFPs in beta (C2) and gamma (C3) frequency ranges. Latency time **(D)** and normalized rate **(E)** for MUA, recorded in PB during ‘PW’ and ‘PW+AWs’ stimulation types. On each box the central mark indicates the median, and the bottom and top edges of the box indicate the 25th and 75th percentiles, respectively. The whiskers extend to the most extreme data points not considered outliers. **(F)** Power spectral density for evoked MUA recorded in B1 (red) and C2 (blue) barrels of the experiment shown on **(C)** (left plot). CI is shown by the shaded area. Group data of the MUA spectral peaks detected in beta and gamma frequency ranges in the recorded barrels, receiving sensory input during ‘PW+AWs’ simulation (right plot). Detected peaks in the beta and gamma frequency ranges of single experiments are shown by circles, whereas mean values and CI for phase and frequency distributions are shown by black crossed lines; **(G)** MUA to LFP coherence for the B1 and C2 barrels for the experiment shown on **(C)** (left), and group data for MUA to LFP coherence peaks in beta and gamma frequency ranges in the recorded barrels, receiving sensory input during ‘PW+AWs’ simulation (right). Detected peaks in the beta and gamma frequency ranges of single experiments are shown by circles, whereas mean values and CI for coherence and frequency distributions are shown by black crossed lines.

During the critical period of barrel map formation topographical sensory input evokes EGO spatially restricted to the corresponding barrel (Minlebaev et al., 2011; Suchkov et al., 2016). However, in natural conditions, the neonatal rat pup predominantly experiences simultaneous deflections of many whiskers either as a result of passive stimulation by the littermates and mother or through a sensory feedback resulting from synchronous multiple whisker movements (Akhmetshina et al., 2016). We sought to characterize evoked cortical responses following multiple whisker stimulation in in several barrels of the barrel cortex in the head-restrained neonatal rat. Short lasting air puffs were applied to the whisker pad to shift vibrissa in the rostrocaudal direction (**Figure 1A**).

Using OIS, we first detected principal and AB positions and characterized evoked OIS following PW+AWs stimulation. During PW+AWs stimulation video recording over the barrels cortex displayed OIS response that covered the large cortical territory (1.73 ± 0.39 mm^2^), while PW stimulations evoked OIS of 0.12 ± 0.03 mm^2^ (*n* = 5, P6-7). In both types of stimulation, OIS started shortly after the stimulation and reached peak responding at 16.4 ± 2.8 s (PW+AWs) and 12.9 ± 3.5 s (PW) which did not differ significantly (*n* = 5, P6-7, *p* > 0.05). The amplitudes of evoked OISs using different types of stimulation (PW and PW+AWs) were not significantly different (mean value 0.18 ± 0.03 and 0.20 ± 0.02%, respectively, *n* = 5, P6-7, **Figure 1B** and see **Supplementary Figures S1A,A2**), supposing the identical metabolic cost for the neuronal activity evoked by different types of stimulation. Using barrels coordinates, we then performed electrophysiological recordings. In agreement with previous data, PW stimulation evoked short latency EGOs (33 ± 5 ms, *n* = 15, P4-7) spatially restricted to the corresponding barrel, since we observed no evoked oscillation in the adjacent barrel. The dominant frequency of EGO was in the gamma band (48 ± 3 Hz with a power of 0.54 ± 0.12 a.u., *n* = 15, P4-7), however, we did observe the peak in the beta frequency range as often (19 ± 3 Hz with power 0.31 ± 0.05 a.u., *n* = 15, P4-7).

**FIGURE 2:**
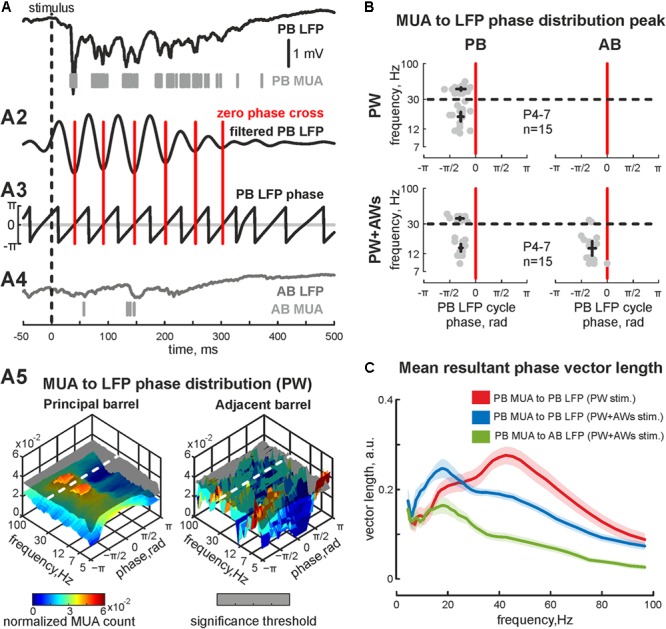
Phase-lock analysis of evoked MUA to LFP in the neonatal rat barrel cortex. **(A)** Evoked LFP (trace) and MUA (bars) recorded in PB following the ‘PW’ stimulation; (A2) wavelet filtered LFP (Morlet mother wavelet with scale coefficient equal to 20 Hz) and (A3) LFP phases (calculated using Hilbert transform) are shown. Red vertical lines correspond to phase 0 (LFP trough). (A4) Simultaneously recorded AB LFP (gray trace) and corresponded AB MUA (gray bars). (A5)3D plot of PB MUA (left) and AB MUA (right) phase lock to PB wavelet filtered LFP during ‘PW’ stimulation. Shaded surfaces correspond to significance threshold. **(B)** Group data of PB (left column) and AB MUA (right column) phase locks to PB wavelet filtered LFP during ‘PW’ (upper row) and ‘PW+AWs’ (lower row) types of stimulation. Detected peaks in the beta and gamma frequency ranges of single experiments are shown by circles, whereas mean values and CI for phase and frequency distributions are shown by black crossed lines; **(C)** group data of resultant vector lengths for PB MUA phase lock to PB LFP at different frequencies during ‘PW’ (red) and ‘PW+AWs’ (blue) types of stimulation. Resultant vector length for cross phase lock of PB MUA to AB LFP at different frequencies during ‘PW+AWs’ stimulation is shown by green color. CI is shown by the shaded area.

PW+AWs stimulation also elicited an oscillatory response that began 34 ± 1 ms after the air-puff and lasted 427 ± 85 ms (*n* = 15, P4-7 neonatal rats, **Figures 1C,D** and see **Supplementary Figure S1C**). Cortical responses during PW+AWs stimulation largely induced a similar number of spikes as PW stimulation (mean value 89 ± 17% of PW MUA rate, **Figure 1E** and see **Supplementary Figures S1B,B2**). The spectral analysis showed the dominant oscillatory frequency ranged in SB frequency with the peak at 16 ± 1 Hz for the experiment shown in **Figure 1C** (group data 17 ± 2 Hz, *n* = 15, P4-7, **Figure 1F** and see **Supplementary Figure S1E**); however gamma frequency oscillations also contributed to evoked oscillatory activity (peak frequency at 40 ± 3 Hz for the experiment shown on **Figure 1C**, while averaged peak frequency was at 50 ± 2 Hz, *n* = 15, P4-7). Estimation of MUA to LFP coherence indicated maximal synchronization of MUA discharges to LFP deflections in the SB and EGO frequency bands (coherence coefficients 0.90 ± 0.02 and 0.89 ± 0.04 for frequencies of 20 ± 2 and 45 ± 4 Hz, respectively, *n* = 15; P4-7, **Figure 1G**). During ‘PW’ stimulation, MUA in the stimulated barrel was also modulated by LFP oscillation in the same gamma and beta frequency ranges (coherence coefficients 0.88 ± 0.02 and 0.89 ± 0.02 for frequencies of 22 ± 3 and 46 ± 4 Hz, respectively, *n* = 15; P4-7). Using Hilbert transformation of the filtered LFP, we calculated the phase preference of MUA to the troughs of evoked cortical responses (**Figure 2A**). We estimated the significance threshold by calculating the phase lock of shuffled MUA to filtered LFP. As an example, we show in **Figure 2B** the MUA phase lock to LFP for the experiment in **Figure 2A**. During PW stimulation, MUA was phase locked to EGO troughs (mean phase lock was -0.28 ± 0.17 rad with the resultant vector length 0.27 ± 0.02 at 44 ± 5 Hz, *n* = 15, P4-7, **Figures 2B,C**). Surprisingly, we also observed MUA phase modulation in the beta frequency. MUA had a similar phase preference in the SB band of LFP as for EGO (mean phase lock was -0.28 ± 0.11 rad with the resultant vector length 0.20 ± 0.02 at 19 ± 5 Hz, *n* = 15, P4-7, **Figures 2B,C**).

**FIGURE 3:**
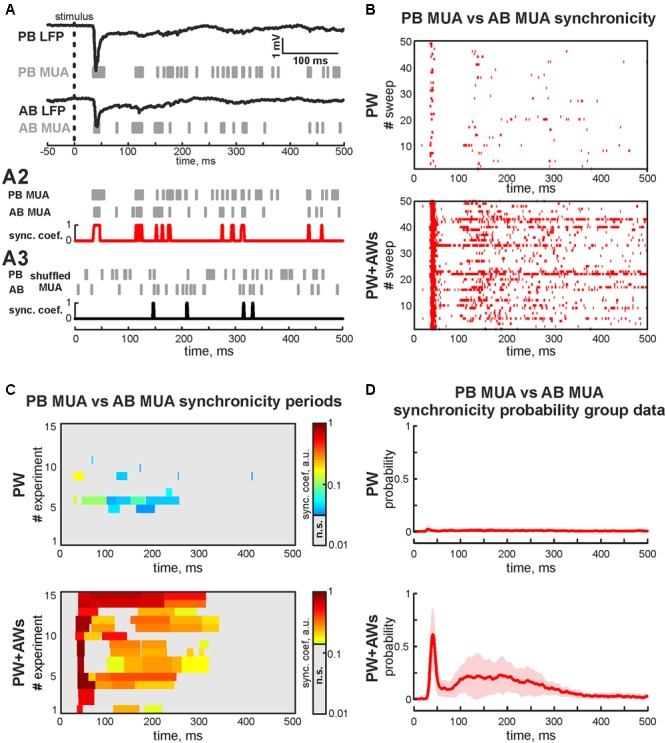
Synchronicity analysis of MUA in adjacent barrels. **(A)** Evoked LFPs (trace) and MUA (bars) recorded in PB and AB during the ‘PW+AWs’ stimulation; (A2) evoked MUA (bars) from PB and AB used for calculation of the synchronicity. Result of the synchronicity analysis is shown by red trace; (A3) shuffled MUA (bars) from PB and AB used for calculation of the threshold of synchronicity significance (black trace); **(B)** post-stimulus time histogram of the synchronous events (red dots) between PB MUA and AB MUA during ‘PW’ (upper box) and ‘PW+AWs’ (lower box) types of stimulation (50 sweep’s analysis is shown). **(C)** Group data of synchronicity analysis between PB MUA and AB MUA for 15 experiments during ‘PW’ (upper box) and ‘PW+AWs’ (lower box) types of stimulation. Synchronicity probability is coded by color. **(D)** Averaged synchronicity probability between PB MUA and AB MUA in the 500 ms window during ‘PW’ (upper plot) or ‘PW+AWs’ (lower plot) types of stimulation. CI is shown by the shaded area.

We also examined whether neurons fired spikes at the preferred phases of LFP during PW+AWs stimulation. MUA predominantly phase locked to troughs of the beta component (mean phase lock was -0.28 ± 0.11 rad with the resultant vector length 0.24 ± 0.02 at 15 ± 3 Hz), while phase locking to the gamma band was less prominent (-0.30 ± 0.12 rad with the resultant vector length 0.19 ± 0.01 at 37 ± 3 Hz, *n* = 15, P4-7, **Figures 2B,C**). Evoked MUA also phase locked to the troughs of the SB component of the LFP in the adjacent barrel (-0.29 ± 0.14 radians with the resultant vector length 0.16 ± 0.02 at 16 ± 8 Hz, *n* = 15, P4-7). In only 2 of 15 experiments, we detected MUA peaks phase locked to LFP between AB in the gamma frequency range (mean phase lock was -0.32 ± 0.04 rad with the resultant vector length 0.11 ± 0.01 at 34 ± 2 Hz, *n* = 2, P6-7). Using the MUA synchronization algorithm, we characterized the duration of synchronicity in evoked responses between recorded barrels **Figure 3A**). MUA between AB during PW stimulation displayed weak synchronicity over evoked responses (synchronization probability 0.05 ± 0.01, mean duration 14 ± 12 ms that was 4 ± 3% of PW evoked answer duration, P4-7, **Figures 3B–D**) in 15 of 15 experiments. PW+AWs type of stimulation induced the long-lasting period of enhanced synchronicity between recorded barrels (synchronization probability 0.43 ± 0.08, duration 221 ± 60 ms, that was 58 ± 20% of evoked answer duration, P4-7, **Figures 3B–D**). So, all whisker stimulation evoked cortical responses with oscillatory components in the SB and EGO frequency ranges. However, the SB component efficiently synchronized cortical activity within and between barrels during an evoked cortical response by whisker stimulation in contrast to EGO component that remained spatially confined to the corresponded barrels.

### Evoked Activity in the Sensory Deprived Barrel

**FIGURE 4:**
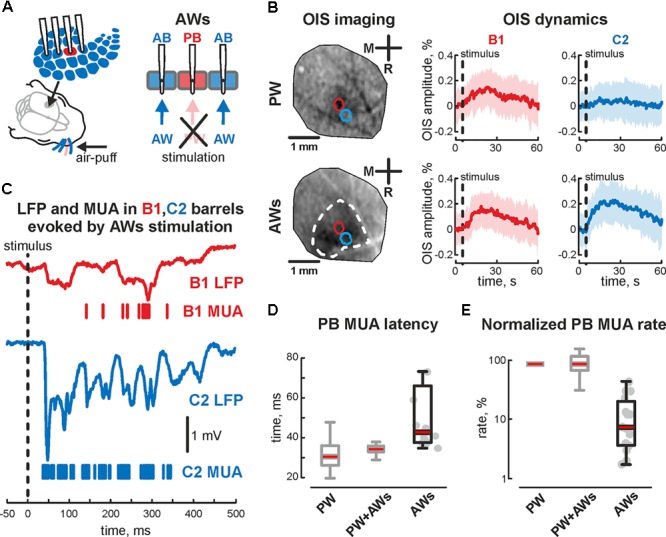
Evoked cortical activity in the sensory deprived barrel during ‘AWs’ stimulation. **(A)** Scheme of the experimental setup with evoked activity recordings from barrel cortex during ‘AWs’ (all except one) whisker stimulation. Note, that PW was pulled out or covered before the experiment. **(B)** OIS imaging during ‘PW’ (upper image) and ‘AWs’ (lower image) types of stimulation. The OIS dynamics in B1 and C2 barrels, during the B1 – ‘PW’ stimulation (upper row) and during the ‘AWs’ (following the sensory deprivation of B1 barrel, lower row) are shown. CI is shown by the shaded area. **(C)** Evoked LFP (trace) and MUA (bars) recorded in B1 (red) and C2 (blue) barrels during ‘AWs’ stimulation. Time latency **(D)** and normalized MUA rate **(E)** recorded in PB during ‘PW,’ ‘PW+AWs,’ and ‘AWs’ types of stimulation. On each box the central mark indicates the median, and the bottom and top edges of the box indicate the 25th and 75th percentiles, respectively. The whiskers extend to the most extreme data points not considered outliers. B1 barrel is sensory deprived. Gray colored boxplots correspond to the data already shown on **Figure 1**.

**FIGURE 5:**
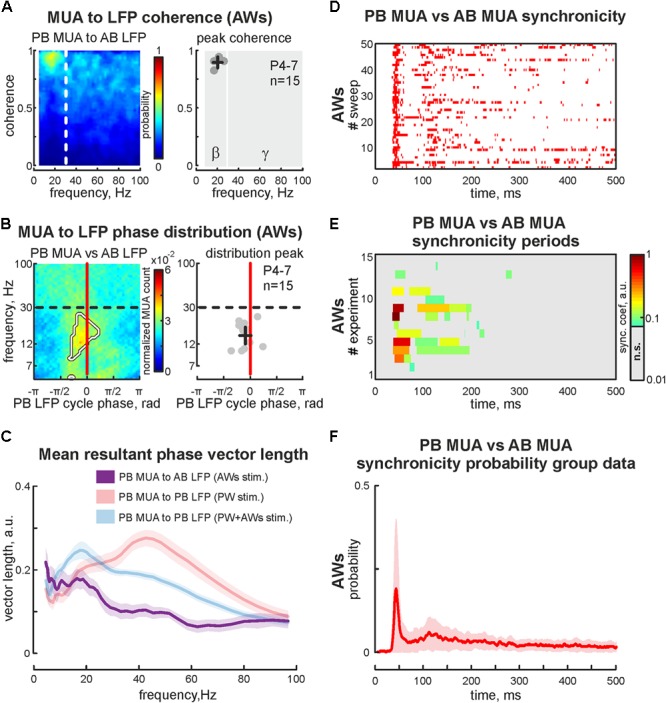
Cross analysis of the cortical activity in sensory deprived and intact barrels during ‘AWs’ stimulation. **(A)** PB MUA to AB LFP coherence (for the experiment presented in **Figure 4C**) is shown on the left. Group data of peak values of the PB MUA to AB LFP coherence in the beta and gamma frequency ranges shown on the right. Detected peaks in the beta and gamma frequency ranges of single experiments are shown by circles, whereas mean values and CI for coherence and frequency distributions are shown by black crossed lines. Note, significantly detectable peaks in the gamma frequency range are absent. **(B)** Phase distribution of PB MUA to AB LFP for experiment shown in **Figure 4C**. The region with significant PB MUA to AB LFP phase locks is circled with a white contour (left plot). Group data of PB MUA to AB LFP phase lock peaks is shown on the right. Detected peaks in the beta and gamma frequency ranges of single experiments are shown by circles, whereas mean values and CI for phase and frequency distributions are shown by black crossed lines. **(C)** Group data of resultant vector lengths for cross phase lock of PB MUA to AB LFP at different frequencies during ‘AWs’ (violet) stimulation. Resultant vector lengths of PB MUA to PB LFP at different frequencies during ‘PW’ (pink), ‘PW+AWs’ (light blue) are shown for comparison (they were already shown in **Figure 2C**). CI is shown by the shaded area. **(D)** Post-stimulus time histogram of the synchronous events (red dots) between PB MUA and AB MUA during ‘AWs’ type of stimulation (50 sweep’s analysis is shown). **(E)** Group data of synchronicity analysis between PB MUA and AB MUA for 15 experiments using ‘AWs’ type of stimulation. Synchronicity probability is coded by color. **(F)** Averaged synchronicity probability between PB MUA and AB MUA in the 500 ms window triggered by the ‘AWs’ stimulation. CI is shown by the shaded area.

The first postnatal week is a period of enhanced plasticity in the barrel cortex (Simons and Land, 1987). Sensory driven activity plays a critical role in cortical map formation (Woolsey and Van der Loos, 1970). Using adult barrel cortex with perinatal sensory deprivation, it was shown that intact sensory input could reinnervate deprived cortical territories (Fox, 1992), which suggests functional reorganization following deprivation. So, we characterized changes in evoked cortical activity associated with acute sensory deprivation. Firstly the positions of 2 barrels (PB and AB) were defined using OIS imaging, followed by the sensory deprivation of one of the identified barrels. For that, we pulled out or covered the whisker projected to barrel called ‘PB.’ Air-puff applied on the whisker pad evoked cortical activity in all but one barrel (AWs type of stimulation, **Figure 4A**). Surprisingly, we observed OIS responses in the sensory deprived barrel. Comparing the features of evoked signals in one barrel before and after sensory deprivation (‘PW’ and ‘AWs’ conditions of stimulation, respectively) showed no differences in OIS amplitude (mean value 0.18 ± 0.03 and 0.14 ± 0.07%, respectively) or peak time (mean value 14.1 ± 2.7 and 11.8 ± 3.8 s, respectively, **Figure 4B**). Presence of OIS in the acute sensory deprived barrel supposes metabolic cost because of the neuronal activity. Using electrophysiological recordings we have found evoked cortical responses following ‘AWs’ stimulation both in the sensory deprived and intact barrels (**Figure 4C**). While oscillatory responses in intact barrels were similar to those observed using PW+AWs stimulation, responses in the deprived barrel were strongly delayed and weaker (mean onset at 46 ± 7 ms and mean PB MUA rate was only 15 ± 7% of PW stimulation, *p* < 0.05, *n* = 15, P4-7, **Figures 4D,E** and see **Supplementary Figures S1B,B2,C**). To avoid the bias inherent in volume conducted LFP spreading into the sensory deprived barrel, we calculated the cross coherence between MUA recorded in the sensory deprived barrel and LFP in the intact one. We found that neuronal discharges in the sensory deprived barrel were strongly modulated by SB oscillatory activity from the intact barrel (cross coherence coefficient 0.89 ± 0.05 at 20 ± 5 Hz, *n* = 15, **Figure 5A**). Moreover, this MUA was also locked to the troughs of SB LFP in the intact barrel (mean phase lock was -0.08 ± 0.18 rad with the resultant vector length 0.17 ± 0.02 at 12 ± 5 Hz, *n* = 15, P4-7, **Figures 5B,C**). Despite low spike rate in 12 of 15 experiments, our analysis of evoked response synchronicity between deprived and intact barrels showed episodes of significant synchronicity that lasted over few SB cycles (mean synchronization coefficient was 0.16 ± 0.06, with mean delay of 43 ± 7 ms after the deflection of the AWs and mean duration of 110 ± 21 ms that corresponds to 15 ± 6% of evoked response duration, P4-7, **Figures 5C,D,F** and see **Supplementary Figure S1D**). These results demonstrated that acute sensory deprived barrel was involved in the sensory evoked cortical response by multiple whisker stimulation. The evoked neuronal discharges in the deprived barrel were synchronized with the neuronal activity in the intact ones by SB oscillation.

## Discussion

Immature cortical activity patterns play the critical role in developing neuronal networks across various species (Kirkby et al., 2013; Ackman and Crair, 2014; Okawa et al., 2014). In the present study, we characterized evoked cortical activities (SB and EGO) in the rodent barrel system following multiple sensory input stimulation to mimic natural conditions. Using simultaneous multishanks extracellular recordings from a thalamorecipient layer of the developing barrel cortex, we found that multiple whisker stimulations evoked complex oscillatory activity composed of EGOs and SBs. Whereas the evoked EGO activity remained within the stimulated barrel, the SB activity was seen over the few recorded barrels, synchronizing neuronal activity in beta frequency. We equally demonstrated the synchronized SB activity in sensory deprived and intact barrels following the multiple whisker stimulation.

The early postnatal weeks comprise a crucial period for cortical maps formation in the rodent somatosensory cortex. Immature cortical activity seems critical to establish proper connectivity and cortical maps during the first postnatal week (Herrmann and Shatz, 1995; Catalano, 1998). Sensory deprivation in the barrel cortex by whisker ablation at birth causes the disappearance of sensory deprived cortical columns and reinnervation of these territories by intact sensory inputs (Woolsey and Van der Loos, 1970; Van der Loos and Woolsey, 1973; Fox, 1995). Moreover, inhibiting cortical activity by the NMDA-R antagonist, D-APV, also alters barrel map plasticity induced by neonatal whisker cauterization (Schlaggar and O’Leary, 1993; Fox et al., 1996; Iwasato et al., 2000) by suppressing neuronal activity needed for cortical maps formation. During neonatal development, the prominent *in vivo* oscillatory activity patterns in the barrel cortex are SBs and EGOs (Khazipov et al., 2004; Hanganu-Opatz et al., 2006; Minlebaev et al., 2007; Colonnese et al., 2010, for review see Kilb et al., 2011). SB and EGO occur so far in primary somatosensory, visual, motor and prefrontal cortices of neonatal rats during the first postnatal week (Khazipov et al., 2004; Hanganu-Opatz et al., 2006; Minlebaev et al., 2007; Colonnese et al., 2010; Brockmann et al., 2011; An et al., 2014; Gerasimova et al., 2014; for review see Kilb et al., 2011). Immature activity patterns are sensory driven, however, inhibiting sensory peripheral activity causes a ∼50% reduction in cortical activity (Khazipov et al., 2004) indicating that neocortical or thalamocortical circuits generate oscillatory activity during the early period of development. While these two oscillatory patterns may co-exist, they can also occur independently. EGOs are best observed during single whisker stimulation but are less prominent during evoked responses by multiple whisker stimulation, where the dominant pattern is SB (Minlebaev et al., 2007, 2009; Yang et al., 2016). Using a combination of single sensory input stimulation and voltage-sensitive dye imaging or multielectrode array recordings, studies demonstrated that SBs and EGOs can synchronize the activity of local neuronal networks (Minlebaev et al., 2011; Yang et al., 2013). Even though EGOs primarily remain within one barrel (Minlebaev et al., 2011), SBs can activate larger cortical territories (200–400 um) (Yang et al., 2009). Moreover, our data suggests that SB could synchronize neuronal activity in neighbor barrels. Beta oscillatory activity *in vitro* was shown to occur in deep cortical layers in the intact preparation of the neonatal mouse (Dupont et al., 2006). Persistent beta activity in the cortical plate requires an intact subplate and assumes that cortical neuronal networks generate beta frequency oscillatory activity (Hanganu et al., 2009; Tolner et al., 2012). Experiments with muscarinic or metabotropic glutamate receptor activation demonstrated a crucial role for NMDA receptors to generate cortical beta frequency oscillatory activity (Dupont et al., 2006). Our results demonstrated delayed synchronized SB activity in the sensory-deprived barrel column, suggesting a multisynaptic excitation necessary for the activity transfer in the sensory deprived column. In contrast to the supragranular layer that is weakly driven by layer 4 during the first postnatal week (Bureau et al., 2004), layer 6 neurons are more mature and could share adult features with axonal projections over many barrel columns to mediate the interbarrel interactions in the infragranular layer (Zhang and Deschênes, 1997). Moreover, layer 6A pyramidal cells that exclusively project to thalamic ventroposterior nucleus have also intracolumnar axon collaterals terminating in layer 4 (Kumar and Ohana, 2008). Therefore, we assume that infragranular layers could be involved in this interbarrel synchronization via SB activity. However, the corticothalamic mechanism of low frequency oscillation synchronization between the barrels is also possible. The mechanisms of sleep spindles synchronization over the large cortical area (Contreras et al., 1997) could be also effective in the neonatal brain since the thalamic synapses implicated in spindle activity are shown to be functional shortly after birth (Evrard and Ropert, 2009). These proposed models of cortical activity synchronization via SB are not incompatible and both thalamic and cortical synchronization mechanisms may coexist in the developing barrel system. *In vivo* and *in vitro* studies provide evidences that immature oscillations are primarily driven by the subcortical structures. EGOs are shown to be elicited by the electrical stimulation of the thalamic nucleus (Yang et al., 2013), whereas little is known about the origin and mechanisms underlying the SBs. Our results are in a good agreement with previous observations, furthermore, demonstration of the SB synchronized neuronal activity between the acute sensory deprived and intact barrels supports the cortical mechanisms of SB generation. Anatomical studies provide clear evidence for a one-to-one relationship between single whiskers and the corresponding neuronal assemblies of relay stations in the barrel system. So, spatial restriction and the absence of synchronicity between EGOs from neighboring barrels are crucial to strengthen the thalamocortical connections between topographically aligned thalamic barreloid and cortical barrel. While the functional role of SB is still debatable, it is clearly demonstrated that subplate selective removal abolishes SBs and prevents the development of the characteristic barrel cortex organization (Tolner et al., 2012). This finding indicates a pivotal role for SBs in the anatomical and functional organization of the barrel cortex. Recent studies in the adult visual cortex reported that sensory signals are conveyed by feedforward gamma activity, which is the substrate for modulation by feedback beta oscillations in a behavioral context (Bastos et al., 2015). This beta activity precedes the enhancement of gamma activity (Richter et al., 2017). So, SB could provide a modulatory role for EGO and thus jointly participate to develop topographic connectivity in the developing somatosensory system.

Both immature patterns are transient and only persist in the rodent’s somatosensory cortex during the first postnatal week. This period of cortical maturation corresponds to the last trimester in human gestation (Clancy et al., 2001; Khazipov et al., 2001). Cortical activity during this period in human premature babies is characterized by characteristic spindle-like oscillations nested in delta waves, termed delta-brush (DB) (Milh et al., 2007; Vecchierini et al., 2007; Koolen et al., 2016). Recently, the auditory cortex of a premature human baby displayed audio click driven gamma activity (Kaminska et al., 2017). Thus, human immature patterns (gamma activity and DB) share common features with EGO and SB. Since DB and gamma activity are the physiological hallmarks of human cerebral cortex development during the late prenatal period (Nevalainen et al., 2015; Kaminska et al., 2017), we assume that impaired human activity patterns during cerebral cortex maturation could cause developmental cognitive and neurological disorders.

## Author Contributions

DS and LS: contributed in acquisition of data and analysis. MM: contributed in supervision and manuscript writing.

## Conflict of Interest Statement

The authors declare that the research was conducted in the absence of any commercial or financial relationships that could be construed as a potential conflict of interest. The reviewer WK and the handling editor declared their shared affiliation.

## Supplementary Material

FIGURE S1 Group data for peak amplitude of the OIS recorded in PB and AB during ‘PW,’ ‘PW+AWs,’ and ‘AWs’ types of stimulation. Statistical comparison between different stimulation types and recordings positions is shown on **(A2)**. **(B)** Group data for normalized MUA recorded in PB and AB during ‘PW,’ ‘PW+AWs,’ and ‘AWs’ types of stimulation. Statistical comparison between different stimulation types and recordings positions is shown on **(B2)**. **(C)** Group data for latency time of the evoked MUA recorded in PB and AB during ‘PW,’ ‘PW+AWs,’ and ‘AWs’ types of stimulation. **(D)** Group data for synchronicity latency time between PB and AB during ‘AWs’ and ‘PW+AWs’ types of stimulation; **(E)** mean values and CI for power and frequency for MUA spectral peaks distributions of the evoked answer in the PB during ‘PW’ (red), ‘PW+AWs’ (blue), and ‘AWs’ (violet) types of stimulation; on each box in **(A–D)** the central mark indicates the median, and the bottom and top edges of the box indicate the 25th and 75th percentiles, respectively. The whiskers extend to the most extreme data points not considered outliers. ^∗∗^Corresponds to significance level < 0.01, while ^∗∗∗^ is *p* < 0.001.
