# Supplementary figures and images for: Horizontal Synchronization of Neuronal Activity in the Barrel Cortex of the Neonatal Rat by Spindle-Burst Oscillations
Source: Front Cell Neurosci. 2018 Jan 19;12:5. doi: 10.3389/fncel.2018.00005 (PMC5780442; doi:10.3389/fncel.2018.00005)

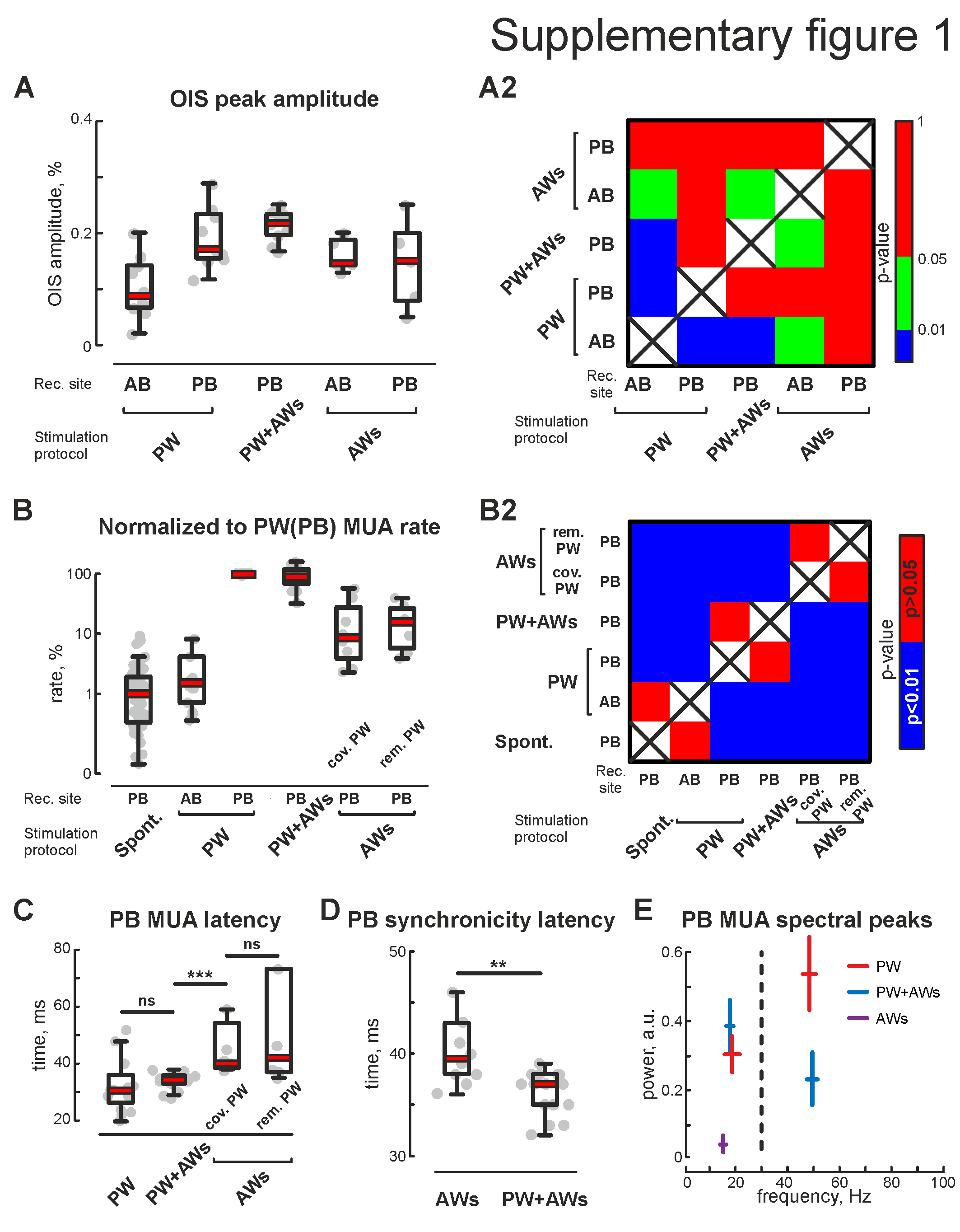

Supplement: FIGURE S1 — Group data for peak amplitude of the OIS recorded in PB and AB during ‘PW,’ ‘PW+AWs,’ and ‘AWs’ types of stimulation. Statistical comparison between different stimulation types and recordings positions is shown on (A2). (B) Group data for normalized MUA recorded in PB and AB during ‘PW,’ ‘PW+AWs,’ and ‘AWs’ types of stimulation. Statistical comparison between different stimulation types and recordings positions is shown on (B2). (C) Group data for latency time of the evoked MUA recorded in PB and AB during ‘PW,’ ‘PW+AWs,’ and ‘AWs’ types of stimulation. (D) Group data for synchronicity latency time between PB and AB during ‘AWs’ and ‘PW+AWs’ types of stimulation; (E) mean values and CI for power and frequency for MUA spectral peaks distributions of the evoked answer in the PB during ‘PW’ (red), ‘PW+AWs’ (blue), and ‘AWs’ (violet) types of stimulation; on each box in (A–D) the central mark indicates the median, and the bottom and top edges of the box indicate the 25th and 75th percentiles, respectively. The whiskers extend to the most extreme data points not considered outliers. ∗∗Corresponds to significance level < 0.01, while ∗∗∗ is p < 0.001. [file Image_1.tif]
